# Glutathione Restores Hg-Induced Morpho-Physiological Retardations by Inducing Phytochelatin and Oxidative Defense in Alfalfa

**DOI:** 10.3390/biology9110364

**Published:** 2020-10-28

**Authors:** Md Atikur Rahman, Ahmad Humayan Kabir, Abul Mandal, Swapan Kumar Roy, Yowook Song, Hee Chung Ji, Ki-Won Lee

**Affiliations:** 1 Grassland and Forage Division, National Institute of Animal Science, Rural Development Administration, Cheonan 31000, Korea; atikbt@korea.kr (M.A.R.); Songs0806@korea.kr (Y.S.); cornhc@korea.kr (H.C.J.); 2 Molecular Plant Physiology Laboratory, Department of Botany, University of Rajshahi, Rajshahi 6205, Bangladesh; ahmad.kabir@ru.ac.bd; 3 Systems Biology Research Center, School of Biosciences, University of Skövde, 54128 Skovde, Sweden; abul.mandal@his.se; 4 Department of Crop Science, Chungbuk National University, Cheongju 28644, Korea; swapankhulna@gmail.com

**Keywords:** glutathione, phytochelatin, ROS scavengers, mercury detoxification, alfalfa

## Abstract

**Simple Summary:**

An ecofriendly approach to mitigate mercury (Hg) toxicity in alfalfa, one of the important forage crops, is highly desirable for environmental sustainability. In this study, the exogenous glutathione (GSH) substantially improved the morphological hindrance and photosynthesis inefficiency in Hg-exposed alfalfa plants. In addition, the Fe and S status of Cd-toxic alfalfa was restored due to GSH supplementation. Interestingly, GSH applied to Hg-exposed plants showed elevated Hg concentration in roots resulted in a substantial deposition of Hg in the root cell wall due to the upregulation of *MsPCS1* and *MsGSH1* genes in roots. It implies that GSH induces PC accumulation in roots enabling excess Hg bound to the cell wall, thereby limiting the transport of Hg to the aerial part of alfalfa. In silico analysis further suggests a conserved motif linked to the phytochelatin synthase domain (CL0125). In addition, GSH induced the GSH concentration and GR activity in protecting alfalfa plants from Hg-induced oxidative damage. These findings can be useful to formulate GSH-based fertilizer or to develop Hg-tolerant alfalfa plants.

**Abstract:**

Mercury (Hg) is toxic to plants, but the effect of glutathione in Hg alleviation was never studied in alfalfa, an important forage crop. In this study, Hg toxicity showed morphological retardation, chlorophyll reduction, and PSII inefficiency, which was restored due to GSH supplementation in alfalfa plants treated with Hg. Results showed a significant increase of Hg, but Fe and S concentrations substantially decreased in root and shoot accompanied by the downregulation of Fe (*MsIRT1*) and S (*MsSultr1;2* and *MsSultr1;3*) transporters in roots of Hg-toxic alfalfa. However, GSH caused a significant decrease of Hg in the shoot, while the root Hg level substantially increased, accompanied by the restoration of Fe and S status, relative to Hg-stressed alfalfa. The subcellular analysis showed a substantial deposition of Hg in the root cell wall accompanied by the increased GSH and PC and the upregulation of *MsPCS1* and *MsGSH1* genes in roots. It suggests the involvement of GSH in triggering PC accumulation, causing excess Hg bound to the cell wall of the root, thereby reducing Hg translocation in alfalfa. Bioinformatics analysis showed that the *MsPCS1* protein demonstrated one common conserved motif linked to the phytochelatin synthase domain (CL0125) with *MtPCS1* and *AtMCS1* homologs. These in silico analysis further confirmed the detoxification role of *MsPCS1* induced by GSH in Hg-toxic alfalfa. Additionally, GSH induces GSH and GR activity to counteract oxidative injuries provoked by Hg-induced H_2_O_2_ and lipid peroxidation. These findings may provide valuable knowledge to popularize GSH-derived fertilizer or to develop Hg-free alfalfa or other forage plants.

## 1. Introduction

Mercury (Hg) toxicity is a severe threat to the plant, soil, and environment due to its bioaccumulation and biomagnification in different ecosystems [1]. The Hg is non-degradable and mobile because of the volatile potentialities of its compounds. Moreover, Hg can be transported within air masses over very long distances [2]. Excess Hg in the environment is linked to several anthropogenic activities, such as mining, sewage sludge, and application of Hg-containing fertilizer and fungicides to soils [3]. Metals exist either as separate entities or in combination with other soil components. Like other metals, Hg may be present in the soil in various forms, among which plants can readily accumulate mercuric ion (Hg^2+^) from the soils [4]. Several studies have shown that Hg can be readily accumulated in higher plants [5]. Low levels of mercuric ion (Hg^2+^) do not significantly affect plant growth, but the excess Hg^2+^ causes many physiological disorders and growth retardation [6,7]. This is undoubtedly a concern for the forage crops as toxic Hg may pass through the animals to the human food chain. The Hg can also adversely affect stomatal movement, water flow, mitochondrial activity, and photosynthesis in plants [8].

To cope with an exposure to the metal(loids) toxicity levels, plants have evolved complex defense strategies like metal exclusion, metal binding in cell wall/vacuole, chelation, and metal sequestrations, along with reactive oxygen species (ROS) homeostasis in cells [6]. Plants can accelerate some low molecular weight thiols like glutathione (GSH) and cysteine (Cys), which possess a high affinity to toxic metals [9]. GSH is a tri-peptide thiol (γ-glutamate-cysteine-glycine), involved in growth and development, cellular defense, and antioxidant defense in stress-induced plants [6]. GSH is a component of reduced sulfur and a precursor of phytochelatins (PCs). In the cytosol, PCs form complexes with toxic metals, subsequently shifted metals into the vacuoles, protect cells from metal-induced toxicity [10]. Several studies documented that PC is involved in metal toxicity alleviation and metal transportation in plants [6,11,12]. PCs are synthesized by reduced glutathione (GSH) catalysis in the presence of the phytochelatin synthase. Phytochelatin synthase genes (*PCS1* and *PCS2*) were found to be expressed differentially [13] and activated by different metal ions in several plants, including *Arabidopsis* [14], *Lotus japonicas* [15], and *Triticum aestivum* [16]. In *Arabidopsis*, the exogenous GSH inhibited Hg entry into root cells by binding to excess Hg [6]. In addition, GSH found to be useful for eliminating Cd [17,18] and As [19] toxicity in several plant species. However, there is no report available so far on the role of GSH in Hg mitigation in forage crops.

Plants exposed to metal toxicity suffer from oxidative stress due to the elevated reactive oxygen species (ROS) such as hydrogen peroxide (H_2_O_2_), superoxide anion (O_2_^•–^)^–^, and hydroxyl radicle (•OH) in cells [20]. Oxidative stress leads to cellular injury, lipid peroxidation, and membrane deterioration in plant cells [21]. However, the induction of the antioxidant defense system is a common strategy in plants to counteract ROS. Several antioxidant enzymes such as superoxide dismutase (SOD), ascorbate peroxidase (APX), catalase (CAT), and glutathione reductase (GR) play critical roles to withstand ROS injury in metal-induced plants. However, plants are often incapable of protecting themselves from ROS damage under metal toxicity. Studies reported that GSH is involved in removing H_2_O_2_ through the AsA-GSH cycle under heavy metal stress in plants [22]. Furthermore, the modulation of cellular redox balance by minimizing ROS accumulation was also reported in tomato [23]. Zhou et al. [24] have shown the induction of SOD and APX in roots of alfalfa in counteracting Hg toxicity in alfalfa. However, the association of GSH in ROS scavenging needs to be validated in Hg-treated forage crops.

Alfalfa (*Medicago sativa* L.) is an important forage legume for its values in animal feed, nitrogen-fixing ability, and biofuel potentialities [25]. Although Hg caused toxicity in alfalfa resulted in poor growth and development, the understanding of the mechanistic basis of Hg-detoxification is still vague. In particular, the ecofriendly uses of GSH on Hg detoxification remained utterly unknown in alfalfa. Therefore, we assessed whether GSH does have a beneficial role in alleviating Hg-toxicity in alfalfa. We further investigated how GSH modulates Hg compartmentation and antioxidant defense in alfalfa under Hg stress.

## 2. Materials and Methods

### 2.1. Plant Culture and Treatment

Alfalfa (Medicago sativa L. cv. Vernal) seeds were surface sterilized using ethanol (70%) for 2 min and then washed three times by deionized water. Seeds were then placed in a germination tray for 2 d before transferring to Hoagland nutrient solution (pH 6.0) [26]. In addition to this nutrient solution, Hg and GSH were added for four treatments: control (without Hg and GSH), +Hg (40 μM), +Hg+GSH (40 μM Hg+50 μM GSH), and +GSH (50 μM GSH) as previously described [6,24]. Nine individual replicates of plants were cultivated in a plastic container (4 L) for each treatment in a controlled environment with 14 h (light) and 10 h dark photoperiod (550–560 µmol s^−1^ per µA) at 25 °C. The nutrient solution was replaced in every 4-d interval, and the plants were cultivated for two weeks.

### 2.2. Determination of Growth and Photosynthesis Parameters

The root and shoot lengths of the harvested plants were measured using a metric scale (cm). The root was dried with blotting paper before measuring the fresh weight (FW). The dry weight (DW) of root and shoot were recorded after dried in an oven at 80 °C for 72 h. The relative water content (RWC%) was determined by using the following formula [27]: (fresh weight − dry weight)/(turgid weight − dry weight) × 100.

The chlorophyll score of young trifoliate leaves was measured on the same replicate plants for each treatment before morphological measurement using SPAD (soil plant analysis development) meter (Minolta, Japan) as instructed by Yuan et al. [28]. The SPAD machine calculated the difference across the leaf between the red and the infrared (650–940 nM) and provided a three-digit SPAD value. [29]. Moreover, Fv/Fm (quantum efficiency of photosystem II) was measured in alfalfa young trifoliate leaves using a portable Chlorophyll Fluorometer PAM-2100 (Heinz Walz, Effeltrich, Germany). Plants were placed at dark for 1 h before data measurement.

### 2.3. Determination of Elemental Concentration in Root, Shoot, Vacuole, and Cell Wall

Elemental concentration in plant tissue was determined using the method described previously [30]. Root and shoot of treated plants were excised from the root–shoot transition zone; roots were washed with distilled water at least three times to remove excess Hg and GSH from the root surface. The root and shoot samples were separately dried in a microwave oven at 80 °C for 72 h. Subsequently, dried samples were digested with HNO_3_/HClO_4_ (3:1 *v*/*v*). Elemental (Hg, Fe, and S) concentration in digested solution was determined based on the standard known solution of that specific element by the inductively coupled plasma mass spectrometry (ICP-MS) system (Agilent 7700, ICP-MS). In order to quantify the compartmentalization of Hg in the root cell wall and vacuole, the centrifugation techniques were performed as previously described [31]. Briefly, fresh roots were washed with ddH_2_O for three times. Subsequently, the samples were homogenized with a mortar and pestle (chilled) using 1 mL extraction buffer containing 500 mM sucrose, 50 mM HEPES, 5.0 mM ascorbic acid, 1.0 mM DTT (dithiothreitol), and 1.0% (*w*/*v*) PVP (polyvinylpyrrolidone). The homogenate was sieved using a nylon cloth (10 μm), and the residue on the nylon cloth was washed twice with the same homogenization buffer (1 mL) and considered as the cell wall fraction. The first filtrate was centrifuged for 10 min at 4000 rpm, and the precipitate (pellet) was defined as the fraction of the vacuole. The pellet was dried in a micro-oven at 80 °C. Finally, the Hg concentration of Hg in the cell wall and vacuole were determined using the ICP-MS approach.

### 2.4. Measurement of MDA and H_2_O_2_ Levels

The level of lipid peroxidation was measured based on the accumulation of malondialdehyde (MDA) levels in plant samples. The MDA level in fresh samples was determined according to the method described previously [32]. Of fresh tissue 100 mg was homogenized in 5 mL 0.1% (*w*/*v*) trichloroacetic acid (TCA). The homogenate was centrifuged at 13,000 rpm for 15 min, 0.7 mL of supernatant was placed in a new tube wherein 0.7 mL TCA 20% containing 0.5% (*w*/*v*) TCA was added. The mixture was incubated at 95 °C for 30 min and then kept the tubes on ice for 3 min. The mixture was centrifuged at 13,000 rpm for 15 min, and the absorbance of the supernatant was read at 532 and 600 nm. The subtracted absorbance and a molar extinction coefficient of 155 mM ^−1^ cm ^−1^ were used to determine the MDA concentration. The MDA concentration was calculated as nmol g ^−1^ fresh weight (FW). H_2_O_2_ level in the alfalfa sample was determined as described earlier [33]. The absorbance of the supernatant was read at 410 nm. Finally, the H_2_O_2_ level was calculated with the extinction coefficient 0.28 μmol^−1^ cm^−1^.

### 2.5. RNA Isolation, cDNA Synthesis, and Gene Expression Analysis by qRT-PCR

Total RNA was isolated from the control and treated alfalfa samples using RNeasy^®^ plant mini kit (QIAGEN, Hilden, Germany). Shortly, 0.1g of tissue sample was homogenized with RNA extraction buffer containing 2 M dithiothreitol (DTT) followed by centrifugation (≥12,000 rpm) for 2 min. Total RNA was obtained from the supernatant, and RNA yield was recovered by adding RNase-free water (30-50 μL). RNA quantification was carried out using a microvolume UV/Vis spectrophotometer (UVIS Drop-99, Taiwan). RNA concentration of ≥200 ng/μL was selected for subsequent analysis, cDNA synthesis was carried out with 1 μg of total RNA using cDNA synthesis kit (Bio-Rad, Hercules, California, CA, USA). qRT-PCR was performed by the CFX96 Real-Time system (BIORAD, Hercules, California, CA, USA) for the expression of target genes using gene-specific primers (Supplementary Table S1). The total 20 μL reaction mixture consisted of 10 μL of iQ^TM^ SYBR^®^ Green Supermix, 2 μL of template cDNA, 0.8 μL of forward primer (10 μM), 0.8 μL of reverse primer (10 μM), and 6.4 μL of DEPC treated H_2_O. The PCR system was programed at 95 °C for 30 s, followed by 40 cycles at 95 °C for 5 s, and 60 °C for 30 s. The relative gene expression was analyzed using the dd^−∆Ct^ method [34], using *MsActin* as an internal control. In each treatment for the qRT-PCR experiment, there were three replicates per biological sample.

### 2.6. In Silico Characterization of the MsPCS1 Gene

The phylogeny of *MsPCS1* was assessed and characterized in contrast to the corresponding PC gene of *Medicago trunculata* (*MtPCS1*) and *Arabidopsis thaliana* (*AtPCS1*) based on the protein sequences retrieved from NCBI databases (Supplementary Table S2). Multiple sequence alignments of these three PCS proteins were performed to identify conserved residues by using Clustal Omega. We also added the molecular function of theses based on gene ontology analysis using the InterProScan software tool (https://www.ebi.ac.uk/interpro/search/sequence-search). Furthermore, the five conserved protein motifs of the proteins were characterized by MEME Suite 5.1.1 [35]. We also searched for the motif annotation and protein domains in the Pfam database [36].

### 2.7. Measurement of Antioxidant Enzymes

Plant tissue (100 mg) samples were homogenized with 100 mM potassium phosphate (KP-buffer, pH 7.0). The homogenate was centrifuged at 13,000 rpm for 15 min, and the aliquots of the supernatant were used for the assaying for the activities of SOD, APX, CAT, and GR. SOD activity was measured in a reaction solution of the plant extracts (100 µL) combined with 0.1 mM EDTA, 50 mM NaHCO_3_ (pH 9.8), and 0.6 mM epinephrine [37]. After 4 min, the confirmation of adrenochrome was recorded at 475 nm. For analysis of APX, extracts was mixed with 0.1 mM EDTA, 50 mM KP-buffer (pH 7.0), 0.1 mM H_2_O_2_, 0.5 mM ascorbic acid, and 0.1 mL extraction. The APX activity was calculated using the extinction coefficient of 2.8 mM ^−1^ cm ^−1^ using the optical density (OD) at 290 nm. The activity of CAT was measured using 100 mmol KP-buffer (pH 7.0), 6% H_2_O_2_, and 100 µL plant extract, and absorbance was read at 240 nm (extinction coefficient 0.036 mM^−1^ cm^−1^) at the 30-60 s intervals. Further, 100 μL plant extract was mixed with 0.2 mol KP-buffer (pH 7.0), 1 mM EDTA, 20 mM oxidized glutathione (GSSG), and 0.2 mM NADPH for measuring the GR activity. The reaction began with GSSG and decreased in absorption at 340 nm in the response of NADPH oxidation. The GR activities were ascertained using the extinction coefficient 6.12 mM^−1^ cm^−1^ [38].

### 2.8. Determination of Glutathione (GSH) and Phytochelatins (PCs)

The concentration of GSH and PC was analyzed in roots of alfalfa using Empower3™ software (Waters Corporation, Milford, MA, USA) by high-performance liquid chromatography (HPLC) at 280 and 360 nm with a dual Waters 2489 detector [39,40]. We used a C18 reverse-phase column as gradient conditions using 100% acetonitrile as the mobile phase. The extracts and samples were diluted (100×) and then filtered (0.22 μm Minisart Syringe Filters; Finetech, Taichung, Taiwan) before injection.

### 2.9. Statistical Analysis

All experiments were repeated three times performed in a completely randomized block design. All experiments had three replicates per treatment with three biological replicates. All data related to physiological and molecular experiments were statistically analyzed using the analysis of variance (ANOVA). Duncan’s multiple range test (DMRT) was additionally conducted to categorize the significant differences at *p* ≤ 0.05. We used SPSS 20.0 and GraphPad Prism 8.3.0 for statistical and graphical analyses, respectively. Three independent replications were considered for the analysis.

## 3. Results

### 3.1. Phenotypic and Morphological Features

**Figure 1:**
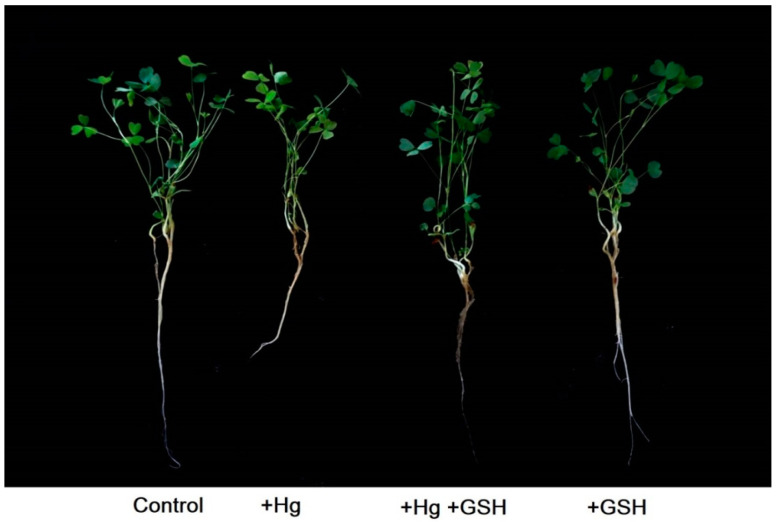
Alfalfa plant phenotypes. Plants cultivated in different conditions of Hg and GSH: +Hg (40 μM), +Hg+GSH (40 μM Hg+50 μM GSH), and +GSH (50 μM GSH).

**Figure 2:**
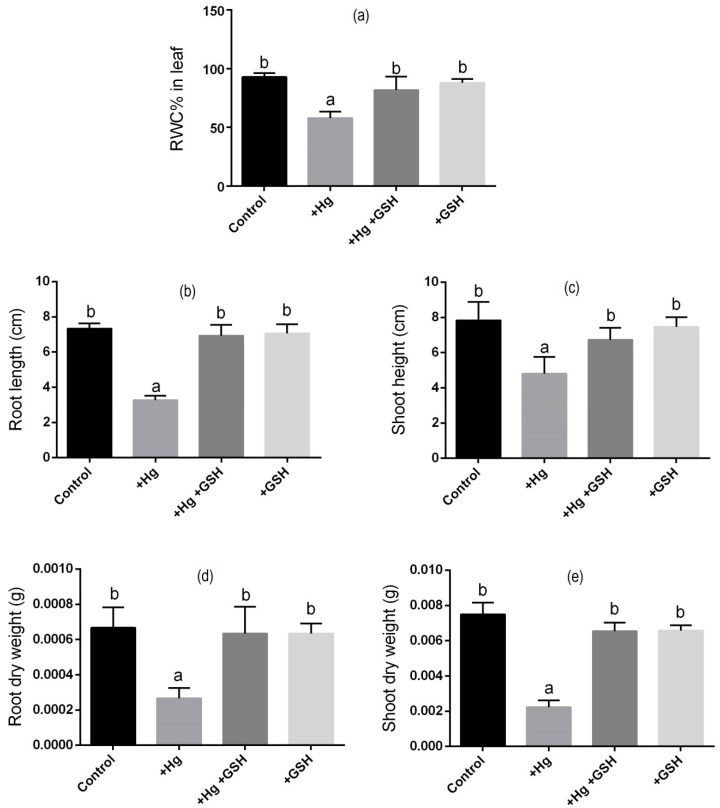
Changes in relative water content (RWC%) (**a**), root length (**b**), shoot height (**c**), root dry weight (**d**), and shoot dry weight (**e**) of alfalfa plants cultivated in different conditions of Hg and GSH: +Hg (40 μM), +Hg+GSH (40 μM Hg+50 μM GSH), and +GSH (50 μM GSH). Data represent means ± SD of three independent biological samples. Different letters indicate a significant difference at the *p* < 0.05 level.

The application of Hg in the solution culture caused phenotypic alterations (Figure 1) and RWC% (Figure 2a) in the leaves of alfalfa relative to the plants grown without any Hg or GSH. The addition of GSH along with Hg showed a similar phenotype and RWC% to that of plants cultivated with or without GSH (control) addition (Figure 2a). Individually, the root and shoot dry weight and their lengths significantly reduced due to Hg stress relative to controls. However, Hg-toxic plants supplemented with exogenous GSH showed a significant improvement in these morphological features, similar to those cultivated solely with GSH or control conditions (Figure 2b,e).

### 3.2. Characterization of Photosynthesis and Metal Elements

**Figure 3:**
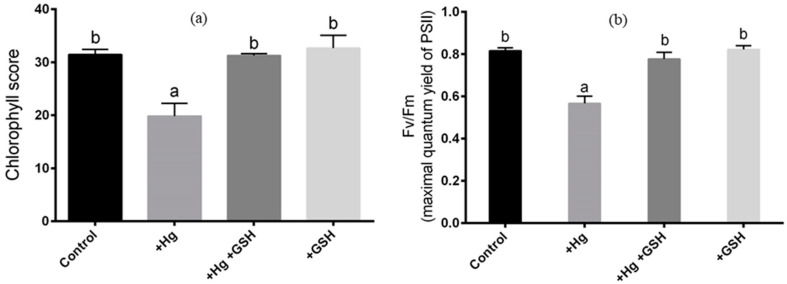
Changes in the chlorophyll score (**a**) and Fv/Fm (**b**) values in young leaves of alfalfa cultivated in different conditions of Hg and GSH: +Hg (40 μM), +Hg+GSH (40 μM Hg+50 μM GSH), and +GSH (50 μM GSH). Data represent means ± SD of three independent biological samples. Different letters indicate a significant difference at the *p* < 0.05 level.

The chlorophyll score and Fv/Fm significantly declined because of Hg stress as opposed to controls. These parameters showed a significant increase in leaves when plants were cultivated with GSH along with Hg compared to Hg-stressed plants (Figure 3a,b). Plant solely cultivated with GSH showed similar chlorophyll score and Fv/Fm values to that of controls or plants treated with Hg and GSH (Figure 3a,b).

**Figure 4:**
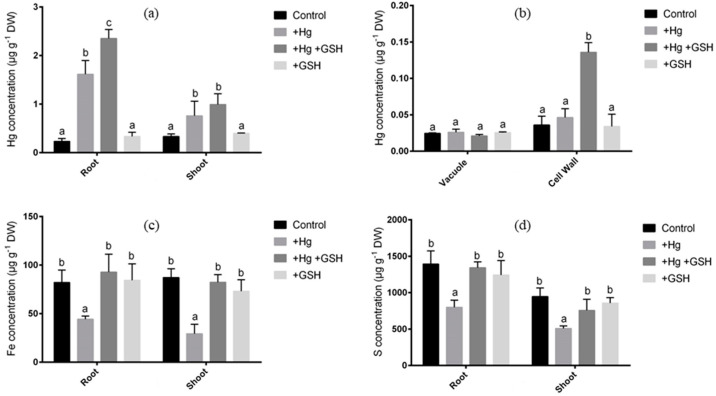
Elemental concentration in root shoot, vacuole, and cell wall of alfalfa. Concentration of Hg (**a**,**b**), Fe (**c**) and S (**d**) in alfalfa cultivated in different conditions of Hg and GSH: +Hg (40 μM), +Hg+GSH (40 μM Hg+50 μM GSH), and +GSH (50 μM GSH). Data represent means ± SD of three independent biological samples. Different letters indicate a significant difference at the *p* < 0.05 level.

ICP-MS analysis showed that the root and shoot of alfalfa showed a significant increase of Hg due to Hg stress relative to non-treated controls (Figure 4a). The surplus of GSH, along with Hg, showed a substantial rise of Hg in roots, while the shoot demonstrated a similar Hg level in contrast to Hg-toxic plants (Figure 4a). Plants solely grown with GSH showed similar Hg levels in root and shoot of alfalfa to that of control plants (Figure 4a). More precisely, the Hg toxicity showed no effect on the concentration of Hg in root vacuole regardless of the treatment conditions (Figure 4b). In the cell wall, the Hg surplus showed no changes in the concentration compared to controls. However, the excess of GSH with Hg showed a significant accumulation of Hg in cell wall relative to the plants cultivated with Hg or GHS or control conditions (Figure 4b). ICP-MS further showed that Fe and S concentration in both root and shoot of alfalfa significantly decreased following Hg surplus relative to untreated controls (Figure 4c,d). The exogenous GSH, along with Hg, caused a significant increase of Fe and S status in both tissues relative to Hg-toxic plants or plants cultivated solely with GSH or untreated conditions (Figure 4c,d).

### 3.3. Expression of Candidate Genes in Roots

**Figure 5:**
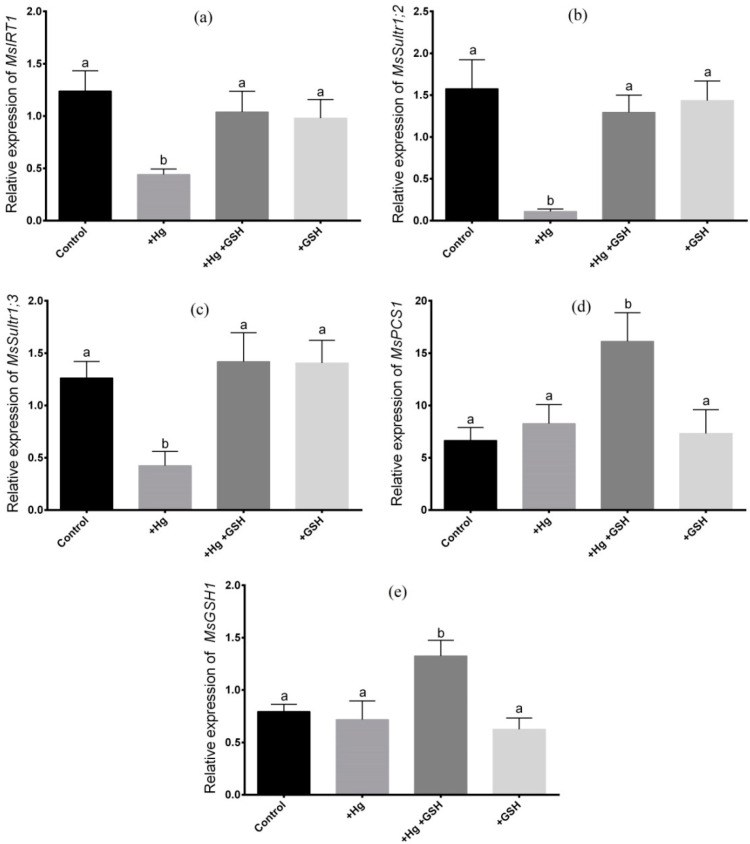
Expression analysis of candidate genes in roots of alfalfa. Relative expression of *MsIRT1* (**a**), *MsSultr1;2* (**b**), *MsSultr1;3* (**c**), *MsPCS1* (**d**) and *MsGSH1*(**e**) in alfalfa cultivated in different conditions of Hg and GSH: +Hg (40 μM), +Hg+GSH (40 μM Hg+50 μM GSH), and +GSH (50 μM GSH). Data represent means ± SD of three independent biological samples. Different letters indicate a significant difference at the *p* < 0.05 level.

The expression of *MsIRT1*, *MsSultr1;2,* and *MsSultr1;3* showed a significant decrease in roots of alfalfa due to Hg toxicity relative to non-treated plants (Figure 5a–c). However, GSH added with Hg showed significant upregulation of these transporter genes in the root compared to Hg-toxic plants, which was similar to the plants grown with or without GSH (Figure 5a–c). The expression of *MsPCS1* and *MsGSH1* showed no changes following Hg supplementation as opposed to controls (Figure 5d,e). However, the expression of *MsPCS1* and *MsGSH1* significantly induced in roots subjected to the dual supplementation of GSH and Hg compared to controls and plants grown under Hg and GSH alone (Figure 5d,e).

### 3.4. In Silico Characterization of the MsPCS1 Gene

**Figure 6:**
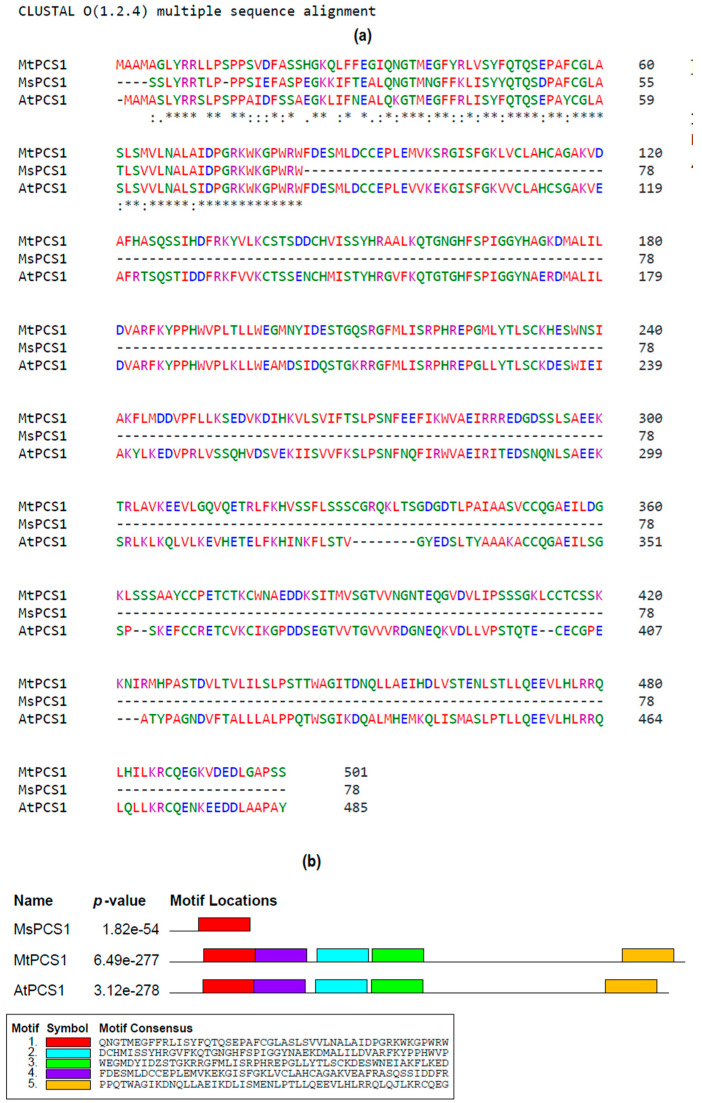
In silico characterization of the *MsPCS1* protein in different bioinformatics platforms. Multiple sequence alignment (**a**) and the motif locations related to the phytochelatin synthase domains (**b**).

MSA showed similarities in the *MsPCS1* protein sequence with *MtPCS1* and *AtPCS1* in 60-119 regions of the protein (Figure 6a). The motif search was performed using the MEME tool that showed one common motif (QNGTMEGFFRLISYFQTQSEPAFCGLASLSVVLNALAIDPGRKWKGPWRW) related to the phytochelatin synthase domain (CL0125) according to the database search (https://myhits.sib.swiss/cgi-bin/motif_scan; Figure 6b).

### 3.5. Changes in Reactive Oxygen Species and Antioxidant Enzymes

**Figure 7:**
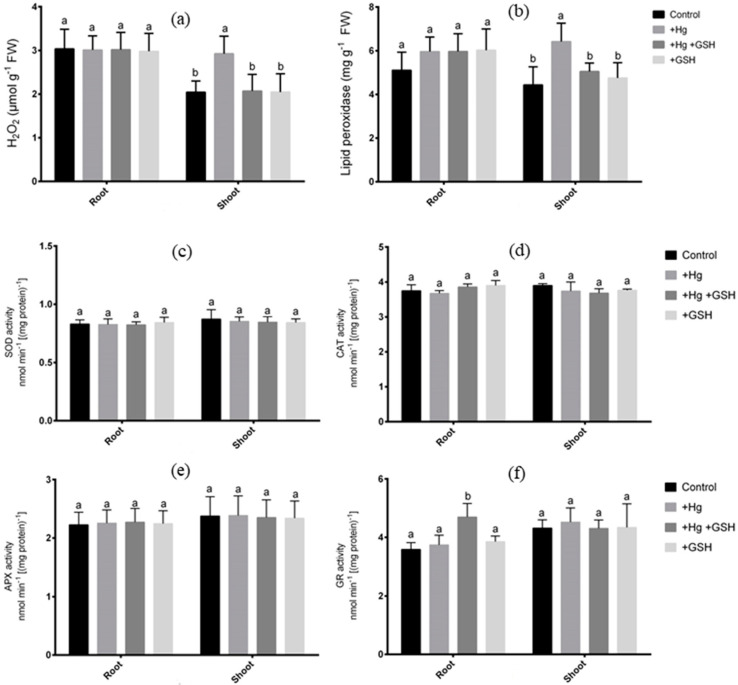
Changes in different ROS indicators and antioxidant enzymes in root and shoot of alfalfa. Response of H_2_O_2_ (**a**), lipid peroxidation (**b**), SOD (**c**), CAT (**d**), APX (**e**) and GR (**f**) in alfalfa cultivated in different conditions of Hg and GSH: +Hg (40 μM), +Hg+GSH (40 μM Hg+50 μM GSH), and +GSH (50 μM GSH). Data represent means ± SD of three independent biological samples. Different letters indicate a significant difference at the *p* < 0.05 level.

In this study, alfalfa plants showed no significant effect of H_2_O_2_ and lipid peroxidase activity in roots in the absence or presence of Hg and GSH (Figure 7a,b). However, the H_2_O_2_ and lipid peroxidase activity significantly increased in the shoot due to Hg surplus relative to controls. The GSH simultaneously applied with Hg showed a significant reduction in these ROS parameters in the shoot relative to Hg-toxic plants. Further, plants solely cultivated with GSH showed similar H_2_O_2_, and lipid peroxidase activity in the shoot to that of controls, and plants are grown in the dual application of Hg and GSH (Figure 7a,b). The enzymatic analysis showed no significant variations in SOD, CAT, and APX activity in either root or shoot of alfalfa in different growth conditions of Hg and GSH (Figure 7c–e). The GR activity only shows a significant increase in roots when plants were cultivated with GSH along with Hg relative to the controls and plants treated with either Hg or GSH alone (Figure 7f). However, the shoot GR activity did not demonstrate any changes regardless of the treatments (Figure 7f).

### 3.6. Changes in Phytochelatin and Glutathione

**Figure 8:**
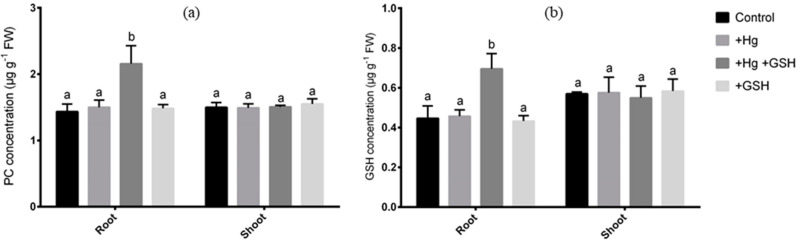
Changes in phytochelatin (PC) and glutathione (GSH) in alfalfa. Concentration of PC (**a**) and GSH (**b**) in the root and shoot of alfalfa cultivated in different conditions of Hg and GSH: +Hg (40 μM), +Hg+GSH (40 μM Hg+50 μM GSH), and +GSH (50 μM GSH). Data represent means ± SD of three independent biological samples. Different letters indicate a significant difference at the *p* < 0.05 level.

Although PC and GSH showed no changes due to Hg stress in roots, the addition of GSH along with Hg caused a significant increase of PC and GSH concentration in roots in contrast to Hg-toxic plants (Figure 8a,b). Plants cultivated solely with GSH showed similar PC and GSH to that of controls and Hg-toxic plants. The alfalfa shoot showed no significant changes in PC and GSH concentration in the absence or presence of Hg or GSH (Figure 8a,b).

## 4. Discussion

Although heavy metals are known to cause growth and metabolic disturbance of plants [6,41], the effect of GSH in Hg detoxification was not yet studied in alfalfa. Along with the stress responses, the sustainable approaches to limit Hg toxicity are highly desirable to ensure plant growth and maintain the natural ecosystem. In this study, GSH exogenously applied to Hg-stressed alfalfa showed dramatic improvement in the morphological and physiological, and cellular status of alfalfa. We further elucidated how GSH activated the detoxification mechanisms of Hg in alfalfa.

### 4.1. Improvement in Morphological and Physiological Features

The Hg is known to cause excess ROS, photosynthesis disturbance, and nutrient imbalance in plants [6,42,43]. In addition, the reduction of plant biomass is an essential indicator of metal-toxic plants. In this study, this was also noticed in alfalfa plants severely affected by Hg stress leading to growth reduction. Similar morphological retardation was also observed in *Pteris vittata* and *Nephrolepis exaltata* due to Hg toxicity [44]. However, we found substantial improvement in root and shoot features treated with GSH in Hg-stressed alfalfa. The morphological recovery is in agreement with the improved RWC in the leaf following GSH addition in the presence of Hg. The change in RWC% is considered to be associated with phytotoxicity in plants [45]. Collectively, this confirms that GSH is a beneficial element in restoring cellular and morphological damages in alfalfa plants subjected to Hg stress.

Phytotoxicity in leaves influences photosynthesis by disturbing both light and dark reactions of photosynthesis [43]. In this study, photosynthetic efficiency was justified by measuring chlorophyll score and Fv/Fm in the presence of Hg and GSH in alfalfa. Moreover, PSII photosynthesis is the primary target for metal toxicity [46]. In this study, chlorophyll synthesis and PSII efficiency showed a close relationship with or without Hg and GSH. The decline in chlorophyll score, along with PSII efficiency, indicates that alfalfa plants were unable to regulate PSII mechanisms under Hg stress. Interestingly, these photosynthesis parameters were fully restored due to GSH, suggesting that GSH facilitates the trapped light in photosynthesis and maintains the PSII activity in response to Hg stress in alfalfa. The Fv/Fm is widely considered as the stress indicator representing PSII photochemistry [47].

Heavy metals itself are toxic, but it also possesses the antagonistic effect of the uptake of other essential elements of plants. In this study, excess Hg showed the inhibitory effect of Fe and S uptake in alfalfa; this may also be correlated with the photosynthetic damages in leaves. The Fe-S cluster is crucial for the structural and physiological functionalities of mitochondria and chloroplasts [48]. The addition GSH caused substantial improvement in Fe and S levels in the root and shoot along with the induction of Fe (*MsIRT1*) and S (*MsSultr1;2* and *MsSultr1;3*) transporters in roots, suggesting the potential role of GSH in restoring Fe and S status in Hg-toxic plants. This might be correlated with the reduced Hg status, thereby arresting Hg’s antagonistic effect in other elements following GSH supply in Hg-exposed alfalfa. Heavy metals alter nutrient absorption and water balance, thereby negatively affecting photosynthesis and development in plants [49]. This implies that the exogenous GSH not only mitigated Hg toxicity but also restored the Fe and S contents when alfalfa plants were grown in Hg-toxic conditions. Further, the improvement of Fe and S due to GSH following Cd stress might also be responsible for the restoration of photosynthetic activity in leaves of Hg-toxic alfalfa.

### 4.2. Mechanisms of Hg Detoxifications

The above-mentioned morphological and physiological improvement indicates that GSH can alleviate Hg-induced toxicity in alfalfa. To get more insight into the mechanisms of detoxification, we performed the metal analysis of Fe and S along with Hg in alfalfa plants. In this study, ICP-MS results showed a significant accumulation of Hg in both the root and shoot of alfalfa under Hg stress. Interestingly, the GSH caused a considerable increase of Hg, even higher than the Hg-toxic plants, in roots, that led to the reduced translocation of Hg into the aerial parts of the plants. It suggests that alfalfa roots retained the excess Hg limiting the detrimental Hg status in leaves due to GSH. Metal compartmentation is a common strategy of hyperaccumulator plants to cope with metal toxicity [50,51]. In this process, the subcellular partitioning of heavy metals coordinated by PC is associated with several plants [39,52]. However, the role of exogenous GSH underlies the detoxification and distribution of Hg is less explored. Cell walls are negatively charged in plants, binding to positively charged metals with the cation exchange ability [53]. The subcellular estimation of Hg showed that GSH-mediated excess Hg accumulation was deposited in the cell wall of roots rather than vacuoles in alfalfa. This led us to determine the concentration of GSH and PC, along with the expression of the respective genes in roots. Results showed a consistent increase of GSH and PC in roots of Hg-toxic alfalfa due to GSH, suggesting that PC might be linked to this cell wall-bound Hg in roots. Consistently, the expression of *MsPCS1* and *MsGSH1* significantly increased when Hg was present in the cultivation media.

The role of *MsPCS1* proteins was further justified with two model plants by bioinformatics analysis. MSA and the phylogenetic tree showed a close relationship of the *MsPCS1* gene with *Medicago* *truncatula* and *Arabidopsis thaliana* homologs, involved with metal binding in plants. Yadav [54], proposed that γ-glutamylcysteine (γ-EC) is exclusively synthesized in the chloroplast. Experimental evidence also reported the expression of *AtPCS1* in chloroplasts [55]. However, it is highly possible that the expression and localization of a particular gene/protein largely depend on the type of magnitude of metal stress. Our wet-lab experiments showed the increased deposition of Hg in the cell wall. The Pfam results showed that all three *PCS1* proteins have the CL0125 domain. Taken together, our findings confirm that GSH involves Hg detoxification in alfalfa plants by inducing PC synthesis that binds to Hg in the root cell wall resulted in decreased Hg accumulation in leaves to stabilize photosynthesis and cellular functions.

### 4.3. Improvement of the Redox Status of the Cells

The generation of oxidative stress causing lipid peroxidation and membrane damages is induced by Hg toxicity in plants [42,56]. Our studies also showed a substantial increase of H_2_O_2_ and lipid peroxidase in the shoot of alfalfa due to Hg toxicity. Although plants do have adaptive features to limit ROS production, this is not often efficient due to toxicity-induced severe cell damage, which is evident in alfalfa. Among the ROS scavengers, high capacity H_2_O_2_ scavengers, GSH, and GR activity were found to be induced Hg-treated alfalfa due to GSH. The participation of GSH and GR activity in H_2_O_2_ scavenging under heavy metal stress was previously reported [57,58]. Interestingly, Zhou et al. [24] reported the stimulation of APX and GR between control and Hg-toxic alfalfa cv. daiyi, which is contradictory to our results in alfalfa cv. Vernal. This implies that the antioxidant responses of different alfalfa cultivars in response to Hg toxicity vary due to different genetic makeup.

The role of exogenous GSH as a ROS scavenger during Hg stress is also supported by a marked reduction in lipid peroxidation, which is increased by Hg stress. The GSH-mediated ROS scavenging has been documented in various transgenic plants having higher endogenous GSH levels [59,60]. Previous studies also suggest that GSH rescues cell vitality and plant growth from Hg stress by relieving Hg-induced oxidative stress in *Arabidopsis*. This study further reported that GSH forms a stable complex with Hg and thus inhibits Hg entry into the *Arabidopsis* plant cell [6]. However, the role of GSH in Hg inhibition was not evident in alfalfa. Rather GSH may, at least partially, allow for ROS scavenging to stabilize redox status in Hg-stressed alfalfa plants.

## 5. Conclusions

This is the first report of GSH uncovering the mechanisms of Hg-detoxification in alfalfa. GSH treated with Hg fully restored the morphological, cellular, and photosynthetic damages in alfalfa. These improvements were accompanied by the reduction of Hg in the shoot due to *MsPCS1*-driven binding of excess Hg in the root cell wall along with the restoration of Fe and S status in Hg-toxic alfalfa plants. In silico analysis showed a close relation of the *MsPCS1* protein with two model plants, sharing a common conserved motif and domain linked to phytochelatin synthesis. In addition, GSH also triggers ROS scavenging through elevated GSH and GR activity in alfalfa. The results revealed that the application of GSH-derived fertilizer could potentially mitigate the toxicity Hg in alfalfa or other forage crops. Furthermore, these findings can be targeted in breeding or transgenic programs to develop plants tolerant to Hg stress.

## Supplementary Materials

The following are available online at https://www.mdpi.com/2079-7737/9/11/364/s1, Table S1: List of primers used in qRT-PCR experiments. Table S2: Accession number and FASTA sequence of *M**sPCS1*, *MtPCS1* and *AtPCS**1* protein homologs.
