# Supplementary material for: Glutathione Restores Hg-Induced Morpho-Physiological Retardations by Inducing Phytochelatin and Oxidative Defense in Alfalfa
Source: Biology (Basel). 2020 Oct 28;9(11):364. doi: 10.3390/biology9110364 (PMC7693861; doi:10.3390/biology9110364)
Supplement: Supplementary file 1 [file biology-09-00364-s001.pdf]

Supplementary Table S1. List of primers used in qRT-PCR experiments.

Gene	Accession Number	Primer	Sequence	Product size
MsActin2	JQ028730.1	Forward	ACCGGTGTGATGGTTGGTAT	173 bp
		Reverse	GCCACACGAAGCTCATTGTA	
MsSULTR1;2	Medtr3g073780	Forward	TATTATCTCCGTGTTGAAGGC	
		Reverse	CAATAAATTTGGCGACCAG	113 bp
MsSULTR1;3	Medtr5g061880	Forward	ATTTATGCCGTCATGGGTAG	101 bp
		Reverse	TTGGGATCAATCTCATTCTGTA	
MsIRT1	XM_003607804.3	Forward	TTTACCCTTGGCGACACGTT	106 bp
		Reverse	CATGAACCCGGTCCCAAGAA	
MsPCS1	AM407892.1	Forward	ACCCTTCCTCCACCTTCAAT	188 bp
		Reverse	CCAGGGTCAATAGCAAGAGC	
MsGSH1	AM411123.1	Forward	GCTGTCAAATGCCCTTCAAT	150 bp
		Reverse	CAAACTCCACAGTCCTGCAA	

Supplementary Table S2. Accession number and FASTA sequence of *MsPCS1, MtPCS1* and *AtPCS1* protein homologs.

Name	Protein accession	FASTA Sequence
MsPCS1	CAL59717.1	SSLYRRTLPPPSIEFASPEGKKIFTEALQNGTMNGFFKLISYYQTQSDPAFCGLATLSV VLNALAIDPGRKWKGPWRW
MtPCS1	XP_013449920.1	MAAMAGLYRRLLPSPPSVDFASSHGKQLFFEGIQNGTMEGFYRLVSYFQTQSEPAF CGLASLSMVLNALAIDPGRKWKGPWRWFDESMLDCCEPLEMVKSRGISFGKLVCL AHCAGAKVDAFHASQSSIHDFRKYVLKCSTSDDCHVISSYHRAALKQTGNGHFSPI GGYHAGKDMALILDVARFKYPPHWVPLTLLWEGMNYIDESTGQSRGFMLISRPHR EPGMLYTLSCKHESWNSIAKFLMDDVPFLLKSEDVKDIHKVLSVIFTSLPSNFEEFIK WVAEIRRREDGDSSLSAEEKTRLAVKEEVLGQVQETRLFKHVSSFLSSSCGRQKLTS GDGDTLPAIAASVCCQGAEILDGKLSSSAAYCCPETCTKCWNAEDDKSITMVSGTV VNGNTEQGDVLIPSSSGKLCCTCSSKKNIRMHPASTDVLTVLILSLPSTTWAGITDNQ LLAEIHDLVSTENLSTLLQEEVLHLRRQLHILKRCQEGKVDEDLGAPSS
AtPCS1	NP_199220.1	MAMASLYRRSLPSPPAIDFSSAEGKLIFNEALQKGTMEGFFRLISYFQTQSEPAYCGL ASLSVVLNALSIDPGRKWKGPWRWFDESMLDCCEPLEVVKEKGISFGKVVCLAHC SGAKVEAFRTSQSTIDDFRKFVVKCTSSENCHMISTYHRGVFKQTGTGHFSPIGGYN AERDMALILDVARFKYPPHWVPLKLLWEAMDSIDQSTGKRRGFMLISRPHREPGLL YTLSCKDESWIEIAKYLKEDVPRLVSSQHVDSVEKIISVVFKSLPSNFNQFIRWVAEI RITEDSNQNLSAEEKSRLKLKQLVLKEVHETELFKHINKFLSTVGYEDSLTYAAAKA CCQGAEILSGSPSKEFCCRETCVKCIKGPDDSEGTVVTGVVVRDGNEQKVDLLVPS TQTECECGPEATYPAGNDVFTALLLALPPQTWSGIKDQALMHEMKQLISMASLPTL LQEEVLHLRRQLQLLKRCQENKEEDDLAAPAY
